# Investigation of the Usefulness of HALP Score in Predicting Short-Term Mortality in Patients with Acute Decompensated Heart Failure in a Coronary Care Unit

**DOI:** 10.3390/medicina60091385

**Published:** 2024-08-24

**Authors:** Rustem Yilmaz, Kenan Toprak, Mustafa Yilmaz, Ahmet Karagoz, Ersoy Öz

**Affiliations:** 1 Department of Cardiology, Faculty of Medicine, Samsun University, Samsun 33805, Turkey; dr.mustafayilmaz@outlook.com.tr (M.Y.); drahmetkgz@hotmail.com (A.K.); 2 Department of Cardiology, Faculty of Medicine, Harran University, Şanlıurfa 63050, Turkey; kentoprak@hotmail.com; 3 Department of Statistics, Yildiz Technical University, Istanbul 34220, Turkey

**Keywords:** acute decompensated heart failure, HALP score, short-term mortality

## Abstract

*Background/Objectives:* Acute decompensated heart failure (ADHF) presents a significant clinical challenge characterized by frequent hospitalizations, high mortality rates, and substantial healthcare costs. The united index of hemoglobin, albumin, lymphocytes and platelets (HALP) is a new indicator that reflects systemic inflammation and nutritional status. This study aimed to investigate the prognostic utility of the HALP score and hematological parameters in predicting short-term mortality among ADHF patients admitted to the coronary care unit (CCU). *Methods:* This investigation adopts a retrospective observational design, encompassing a cohort of patients with ADHF who were followed in the CCU at our medical institution between January 2019 and April 2024. *Results:* The cohort of 227 individuals was dichotomized into two subsets based on the presence or absence of short-term mortality in the hospital, resulting in 163 (71.8%) and 64 (28.2%) individuals in the survivor and exitus groups, respectively. Age was significantly higher in the exitus group (*p*-value = 0.004). Hemoglobin, lymphocyte count, platelet count, albumin, and HALP score were significantly higher in the survivor group (all *p*-values < 0.001). No significant difference was observed between the groups in terms of gender, diabetes mellitus (DM), coronary artery disease (CAD), or ejection fraction (EF), although hypertension (HT) prevalence was significantly higher in the exitus group (*p*-value = 0.038). ROC analysis demonstrated that hemoglobin, lymphocyte, albumin, and HALP score had significant discriminative power, with albumin showing the highest AUC (0.814). *Conclusions:* In conclusion, the HALP score and hematological parameters represent valuable prognostic feature for short-term mortality prediction in ADHF patients admitted to the CCU. These findings underscore the importance of early risk stratification and targeted interventions guided by comprehensive biomarker assessments in optimizing patient outcomes.

## 1. Introduction

Acute decompensated heart failure (ADHF) is characterized by the sudden or gradual onset of symptoms and signs associated with heart failure. The predominant etiology of this condition is the sudden worsening of chronic heart failure. This clinical condition is one of the most challenging clinical scenarios as it requires hospitalization and has the potential to result in mortality [1]. The incidence of patients admitted to coronary care unit (CCU) with the diagnosis of acute decompensated heart failure is increasing every year, and it has become the most common reason for long-term hospitalizations, especially in patients over the age of 65 [2]. Moreover, hospital costs for patients with ADHF are increasing. Prognostic evaluation plays an increasingly important role in the treatment of these patients. The inseparable coexistence of inflammation and heart failure has been known for a long time in several studies [3,4]. Recent studies have reported that albumin, C-reactive protein, and lymphocytes predict the prognosis in patients with ADHF [5,6,7,8].

The HALP score, consisting of four key blood parameters, hemoglobin, albumin, lymphocyte count and platelet count, is a new and easily calculable biomarker-based tool and an available measurement that combines important information about the patient’s nutritional status and systemic inflammation [9,10]. Anemia and hypoalbuminemia are indirect indicators of malnutrition [11]. Lymphocytes are a member of the white cell group that plays an important role in inflammation. Lymphopenia has been associated with poorer survival in patients with heart failure [12]. In addition, high platelets have been shown to be associated with mortality because they increase the risk of thromboembolism and atherosclerotic lesions [13]. Concurrently, the hemoglobin, albumin, lymphocyte, and platelet (HALP) score has emerged as a robust indicator of various clinical outcomes across a spectrum of diseases, including but not limited to, oncological conditions and cardiovascular diseases [14,15,16,17]. In a recent study, HALP score was found to be an important marker in predicting no-reflow phenomenon and short-term mortality in ST-segment elevation myocardial infarction (STEMI) patients who underwent primary percutaneous intervention [18]. In addition, many studies have been conducted using hematological parameters to predict heart failure (HF) prognosis [19,20], Anemia is the most important and independent determinant of HF mortality. In addition, red-cell distribution width (RDW) was found to be associated with mortality independent of anemia [21,22]. Differentiation between low-risk and high-risk patients can reduce unnecessary hospitalizations as well as the economic burden. Therefore, biomarkers and scoring systems that can predict poor prognosis in patients with ADHF may improve patient management and reduce mortality rates.

Current studies are mostly focused on predicting long-term mortality. However, there are few studies in the literature on the usefulness of HALP score and hematological parameters in predicting short-term mortality in patients with ADHF in the CCU. The number of studies regarding the use of HALP score in risk stratification of patients with ADHF is limited. Moreover, the current literature contains information about the parameters of HALP score such as hemoglobin, albumin, lymphocyte and platelets separately. The goal of this study is to investigate the usefulness of HALP score, a combination of these parameters, in predicting short-term mortality in patients with ADHF in the CCU.

## 2. Materials and Methods

### 2.1. Study Design and Dataset

This investigation adopts a retrospective observational design, encompassing a cohort of patients with ADHF who were admitted to CCU at our medical institution between January 2019 and April 2024 (please see Supplementary Table S1 for the whole dataset). This cohort of 227 individuals in total was subsequently dichotomized into two subsets based on the presence or absence of the short-term mortality in the hospital (Exitus Group and Survivor Group). Ethical clearance for this study was duly obtained from the institutional ethics committee, ensuring that the research adheres to established ethical guidelines (Date: 13 March 2024; Protocol number: 2024/6/16, Samsun University, Turkey).

### 2.2. Inclusion and Exclusion Criteria

The patients hospitalized in the CCU with a diagnosis of acute decompensated heart failure were included in this study. 

The exclusion criteria from the study are given below:

(a) Age <18 years and >80 years;
(b) Use of drugs known to affect the complete blood count;
(c) A hemoglobin value of less than 8 g/dL;
(d) Active bleeding;
(e) Severe renal impairment [estimated glomerular filtration rate (eGFR) 15 mL/min/m^2^];
(f) A history of cancer detected within the previous year;
(g) Severe liver impairment;
(h) Presence of missing data about the patient.

### 2.3. Data Collection

Data pertaining to demographic variables, MI localizations, and comorbidities were extracted from electronic medical records. Hematological and biochemical parameters including urea (mg/dL), creatinine, albumin, c-reactive protein, aspartate transaminase, alkaline phosphatase, troponin, hemoglobin, platelet, white blood cell, neutrophil, lymphocyte, basophil, monocytes, and eosinophil counts were ascertained from venous blood samples obtained during the initial emergency department admission.

### 2.4. HALP Score Calculation

The HALP score was calculated using a previously defined standardized formula: Hemoglobin (g/L) × Albumin (g/L) × Lymphocytes (/L)/Platelets (/L), serving as a key variable in the study’s predictive model.

### 2.5. Statistical Analysis

In this study, the Mann–Whitney U test was used to determine whether there was a statistically significant difference between the groups. Chi-Square test was applied for categorical parameters. In addition, correlation analysis was conducted between the parameters. In our statistical analysis, *p*-values were used to determine the significance of the results. A *p*-value less than 0.05 was considered statistically significant, indicating that the observed results are unlikely to have occurred by chance. The area under the curve (AUC) from the ROC analysis was utilized to assess the discriminative ability of the biomarkers. An AUC value ranges from 0.5 (no discrimination) to 1 (perfect discrimination). AUC values greater than 0.7 are generally considered acceptable, while values above 0.8 indicate excellent discriminatory power.

## 3. Results

Table 1 presents a comparison of demographic, hematological and biochemical (parameters between the survivor and exitus groups. 

Age was higher in the exitus group, while hemoglobin, lymphocyte, platelet, albumin, and HALP score were higher in the survivor group. There was no significant difference between survivor and exitus groups in terms of gender, diabetes mellitus (DM), coronary artery disease (CAD) and ejection fraction (EF%), while prevelance of hypertension (HT) was significantly higher in the exitus group. (*p*-value = 0.038). 

**Figure 1:**
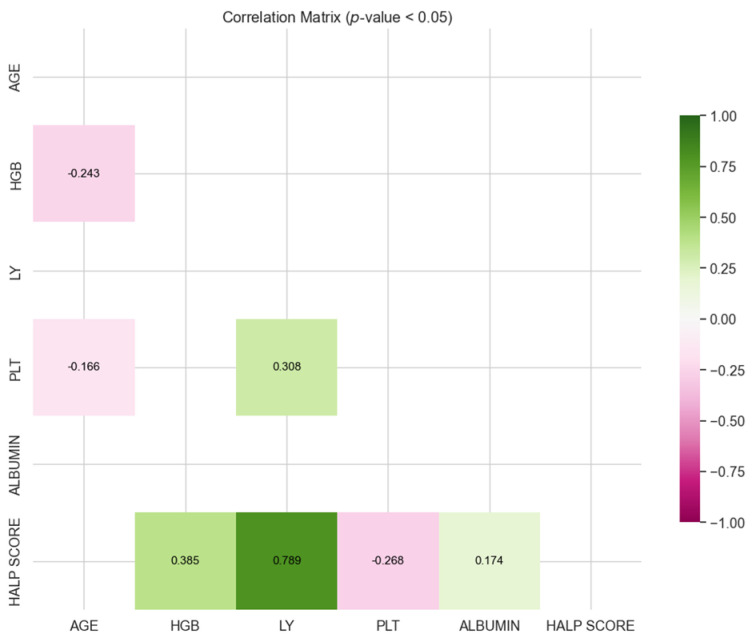
Correlation matrix for survivor group.

**Figure 2:**
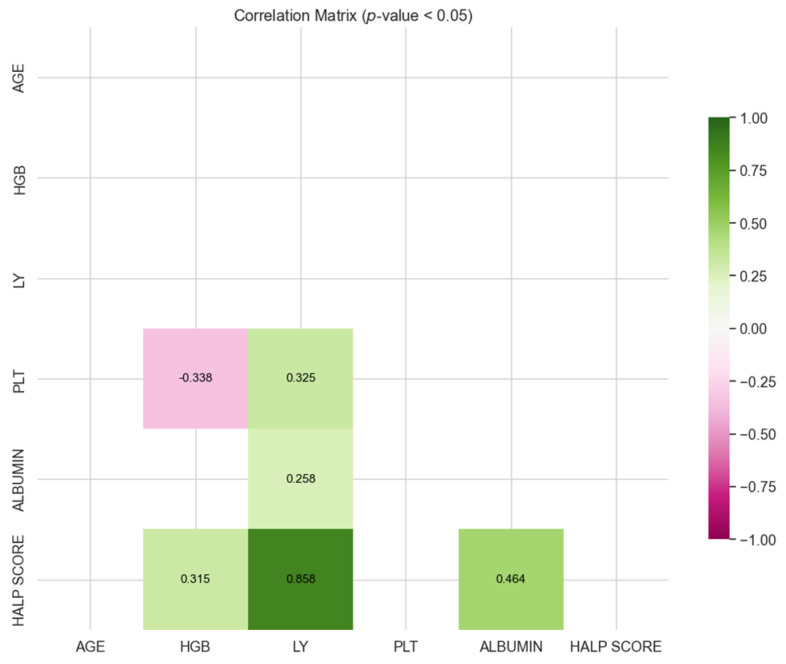
Correlation matrix for exitus group.

Figure 1 and Figure 2 show the correlation coefficients found to be significant for survivor and exitus groups. Positive correlation coefficients indicate the same directional movement between the two parameters (or ratios), while negative correlation coefficients indicate the opposite directional movement.

Figure 1 shows that there was a weak negative correlation between age, platelet and hemoglobin, and a weak positive correlation between platelet and lymphocyte. Additionally, there were significant correlations between HALP score and hemoglobin, lymphocyte, platelet, and albumin. Notably, there was a very strong positive correlation between HALP score and lymphocyte.

Figure 2 shows that there was a weak positive correlation between albumin and lymphocyte, and a moderate negative correlation between platelet and hemoglobin. Similarly, there was a moderate positive correlation between platelet and lymphocyte, between HALP score and hemoglobin, and between HALP score and albumin. Additionally, there was a very strong positive correlation between HALP score and lymphocyte.

**Figure 3:**
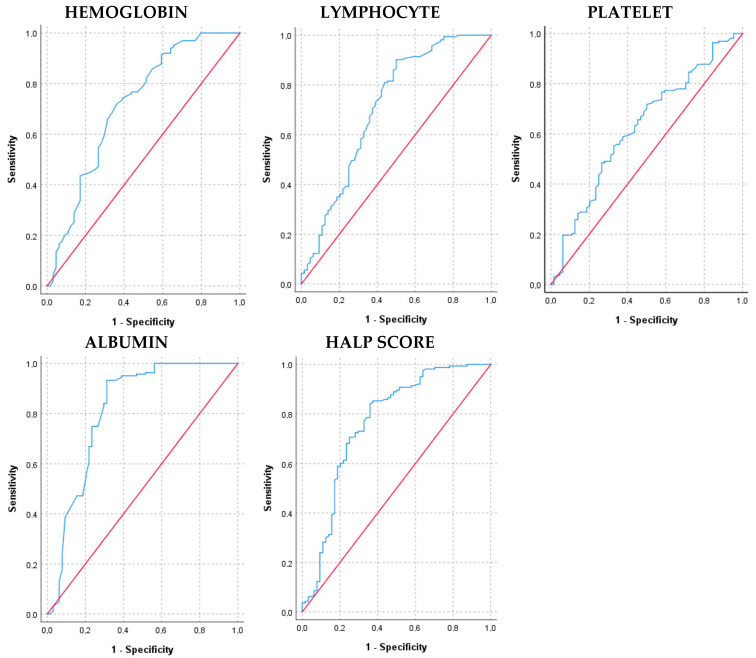
Hemoglobin, lymphocyte, platelet, albumin, and HALP score for survivor and exitus groups are depicted in the ROC curve. The diagonal red line denotes the ROC curve of a random classifier.

The curves obtained in ROC analysis are given in Figure 3, and the ROC curve metrics are given in Table 2.

ROC analysis for hemoglobin revealed an AUC value of 0.714. This value indicates that hemoglobin has a good ability to distinguish between the two groups. Additionally, the *p*-value is <0.001, confirming that hemoglobin is statistically significant in ROC analysis. The cut-off value is 12.95, which is an optimal discriminative value. Sensitivity is 71.8% and specificity is 64.1%. These results indicate that hemoglobin has a moderate level of discrimination between the two groups and can be considered as a reliable biomarker. Hemoglobin performs well, especially in terms of sensitivity. Overall, hemoglobin can be used as a diagnostic tool, but it should be evaluated in conjunction with other parameters.

ROC analysis for lymphocyte revealed an AUC value of 0.709, indicating that lymphocyte has a good ability to distinguish between the two groups. The cut-off value is 1.33, which is an optimal discriminative value. Sensitivity is 90.2% and specificity is 50%. These results indicate that lymphocyte has a moderate level of discrimination between the two groups and can be considered a good biomarker, particularly due to its high sensitivity.

The AUC value for the platelet indicates that platelet has limited ability to distinguish between the two groups. Although platelet value is statistically significant in ROC analysis, the AUC value is low. The cut-off value is 256.5, identified as an optimal discriminative value. Sensitivity is 55.2% and specificity is 67.2%. These results suggest that platelet has limited discrimination power between the two groups.

The AUC value for the albumin indicates that this parameter has a very good ability to discriminate between two groups. AUC values above 0.8 indicate that they have a strong discrimination power. The *p*-value indicates that albumin is statistically significant in the ROC curve analysis. This indicates that the results obtained are not random and that albumin provides significant discrimination. The sensitivity and specificity values of albumin indicate that this parameter has a very good discrimination power between the two groups and is a reliable biomarker. The cut-off value of 36.70 is an optimal discriminative value.

The ROC analysis results for HALP score yielded an AUC value of 0.765. The corresponding *p*-value was found to be <0.001, indicating that HALP score significantly distinguishes between the two groups. An optimal cut-off value of 3.305 was identified. While HALP score demonstrates a high sensitivity in detecting disease, it has limitations in terms of specificity. Overall, HALP score suggests that it could be a valuable biomarker in clinical practice.

The *p*-values associated with the AUC values confirm the statistical significance of the results, providing evidence that the predictive capabilities of the HALP score and other biomarkers are reliable. For example, the HALP score demonstrated a high AUC value of 0.765 with a *p*-value of <0.001, suggesting that it is a strong predictor of short-term mortality in ADHF patients.

## 4. Discussion

Our study results revealed that higher levels of albumin, hemoglobin, lymphocyte, and HALP score are associated with better survival outcomes in ADHF patients. It is essential to interpret the reported *p*-values and AUCs to understand the clinical implications of our findings. The *p*-values <0.001 for hemoglobin, lymphocyte, albumin, and HALP score indicate that the differences observed between the survivor and exitus groups are statistically significant and not due to random variation. The AUC values demonstrate the discriminatory power of these biomarkers, with albumin showing the highest AUC (0.814), followed by HALP score (0.765), hemoglobin (0.714), and lymphocyte (0.709). These AUC values suggest that these biomarkers have good to moderate ability to distinguish between the two groups, with albumin having the strongest discriminative capability. Hence, these parameters can be considered reliable prognostic tools for assessing short-term mortality risk in this patient population. The clinical utility of these findings lies in their potential to guide early and targeted interventions to improve patient outcomes.

Acute decompensated heart failure (ADHF) presents a significant clinical challenge characterized by frequent hospitalizations, high mortality rates, and substantial healthcare costs [23,24]. This study aimed to investigate the prognostic utility of the HALP score and hematological parameters in predicting short-term mortality among ADHF patients admitted to the coronary care unit (CCU). Our findings contribute valuable insights into the predictive capacity of these biomarkers and their potential implications for clinical practice. Our analysis reveals notable distinctions between groups based on key parameters: age levels were higher in the exitus group, whereas the survivor group exhibited elevated levels of hemoglobin, lymphocyte, platelet, albumin, and HALP score. These findings underscore significant biochemical and hematological differences between the compared groups. Additionally, our findings indicate no significant difference between the survivor and exitus groups in terms of gender, DM, CAD, and EF. However, a statistically significant difference was observed for HT, suggesting a significant association between this parameter and group classification.

When the correlation matrices were evaluated together, statistically significant correlations were found between similar parameters in the survivor and exitus groups. For the survivor group, there is a weak negative correlation between age, platelet, and hemoglobin, and a weak positive correlation between platelet and lymphocyte. Additionally, there were significant correlations between HALP score and hemoglobin, lymphocyte, platelet, and albumin, with a notably very strong positive correlation between HALP score and lymphocyte. In contrast, for the exitus group, a weak positive correlation between ALB and lymphocyte, and a moderate negative correlation between platelet and hemoglobin were found. Similarly, there was a moderate positive correlation between platelet and lymphocyte, between HALP score and hemoglobin, and between HALP score and albumin, along with a very strong positive correlation between HALP score and lymphocyte. From the correlations involving HALP score in both groups, it can be concluded that HALP score consistently shows significant relationships with hemoglobin, lymphocyte, platelet, and albumin. Notably, in both groups, there is a very strong positive correlation between HALP score and lymphocyte, indicating a robust and consistent association regardless of the group.

In evaluating the diagnostic potential of various hematological and biochemical markers using ROC analysis, our findings highlight the differing discriminatory capabilities of several key parameters. Among the parameters analyzed, hemoglobin, lymphocyte, platelet, albumin, and HALP score exhibit varying degrees of discriminatory performance. Hemoglobin and lymphocyte show moderate to good discrimination abilities, with hemoglobin performing well in sensitivity and lymphocyte standing out for its high sensitivity despite lower specificity. Platelets, on the other hand, demonstrate limited discrimination power despite being statistically significant. In contrast, albumin displays strong discriminatory power with a high AUC, indicating its robust ability to distinguish between the groups effectively. HALP score also shows significant discriminatory ability with a high AUC and low *p*-value, highlighting its potential as a valuable biomarker, particularly in disease detection scenarios. Integrating these parameters in clinical assessments can enhance diagnostic accuracy and provide valuable insights into disease profiling and patient management strategies. However, understanding their specific strengths and limitations is crucial for their optimal use in clinical practice.

The HALP score, which is derived from four essential blood parameters: Hemoglobin, Albumin, Lymphocyte count, and Platelet count is a newly developed, easily computable biomarker tool. It serves as a valuable metric that integrates critical insights into a patient’s nutritional status and systemic inflammation. In addition, these studies found that a low HALP score was an effective marker for predicting long-term mortality due to cardiovascular diseases in the general population [9,10]. His integrative approach has gained traction in prognostic assessments across various disease states due to its simplicity and comprehensive reflection of physiological derangements [14,15,16,17]. In our study, lower HALP scores were significantly associated with increased short-term mortality risk. This aligns with previous research demonstrating the prognostic value of HALP score in diverse clinical contexts, including cardiovascular diseases and critical care settings [15,16,25]. A recent study found that the HALP score was an important marker for risk stratification and short-term mortality prediction in patients with ST-segment elevation myocardial infarction (STEMI) undergoing primary percutaneous intervention [18]. In another study, the modified HALP score was shown to be an independent prognostic index in patients with acute heart failure [26]. The robust discriminatory ability of HALP score underscores its potential as a reliable tool for risk stratification in ADHF, enabling early identification of patients at heightened risk of adverse outcomes.

Hemoglobin levels emerged as a pivotal predictor in our analysis, with higher levels associated with improved survival outcomes. Anemia, prevalent in heart failure patients, exacerbates myocardial oxygen demand and compromises tissue perfusion, thereby exacerbating cardiac function and contributing to poorer outcomes [21,27,28,29]. The observed association between higher hemoglobin levels and better prognosis highlights the critical role of adequate oxygen-carrying capacity in mitigating the adverse effects of heart failure exacerbations.

Lymphocyte count, another component of the HALP score, demonstrated significant prognostic value in our cohort. Lower lymphocyte counts were strongly correlated with increased mortality risk, indicative of immune dysregulation and heightened inflammatory response in ADHF [30]. A recent study found that in hospital mortality was higher in patients with heart failure and lymphopenia [31]. Lymphocytes play a pivotal role in modulating systemic inflammation and immune surveillance, with depletion suggesting a state of immune exhaustion or dysfunction that exacerbates cardiovascular pathology and contributes to disease progression [30].

Platelet count, while statistically significant in differentiating between survivor and exitus groups, exhibited modest discriminatory power compared to other parameters such as hemoglobin and albumin. Platelets contribute to thrombotic processes and inflammatory cascades implicated in heart failure exacerbations [32,33,34]. In a study conducted on patients admitted to intensive care unit due to acute heart failure, it was found that early increase in baseline platelet count increased in-hospital mortality [35]. The observed associations underscore the multifaceted roles of platelets in cardiovascular pathophysiology, warranting further investigation into their specific contributions to disease progression and outcomes in ADHF.

Albumin, a key marker of nutritional status and inflammation, emerged as a robust predictor of short-term mortality in our study. Lower serum albumin levels were significantly associated with increased mortality risk, reflecting systemic inflammation, malnutrition, and organ dysfunction commonly observed in ADHF patients [36,37]. Hypoalbuminemia serves as a surrogate marker for disease severity and complications, guiding clinical decision-making and prognostic assessments in acute settings.

The ROC analysis reaffirmed the diagnostic performance of these biomarkers, with HALP score demonstrating superior discriminatory ability compared to individual hematological parameters. High sensitivity and specificity of HALP score underscore its potential as a comprehensive prognostic tool, facilitating risk stratification and personalized management strategies in ADHF. The integration of these biomarkers into routine clinical practice could enhance prognostic accuracy, inform therapeutic decisions, and optimize resource allocation in acute care settings.

Our findings highlight the importance of early risk stratification using readily available laboratory parameters, facilitating timely intervention and optimized management strategies for ADHF patients at higher risk of mortality. These biomarkers not only aid in prognostication but also offer insights into the pathophysiological mechanisms underlying disease progression in ADHF. Moving forward, prospective studies are warranted to validate our findings across diverse patient populations and healthcare settings. Further exploration of the mechanistic links between hematological parameters, systemic inflammation, and cardiac function could elucidate novel therapeutic targets aimed at improving outcomes in ADHF. In clinical practice, incorporating HALP score and hematological parameters into routine assessments may enhance clinical decision-making, optimize resource allocation, and ultimately improve patient outcomes in acute decompensated heart failure. For example, a patient with a low HALP score can be given more extensive medical treatment such as larger spectrum antibiotics and stronger anti-inflammatory therapy. Future research endeavors should focus on refining risk stratification models and evaluating the impact of targeted interventions guided by these biomarkers on patient-centered outcomes.

Our study has several limitations. The retrospective observational design limits causal inference and may introduce selection bias despite rigorous adjustment for confounding factors. Future prospective studies with larger, multicenter cohorts are warranted to validate our findings and elucidate the mechanistic underpinnings linking hematological parameters to clinical outcomes in ADHF. Additionally, exploring dynamic changes in biomarker profiles over time could provide further insights into disease progression and therapeutic response.

## 5. Conclusions

In conclusion, the HALP score and hematological parameters represent valuable prognostic markers for short-term mortality prediction in ADHF patients admitted to the CCU. These findings underscore the importance of early risk stratification and targeted interventions guided by comprehensive biomarker assessments in optimizing patient outcomes. Incorporating HALP score and hematological parameters into routine clinical assessments has the potential to enhance prognostic accuracy, refine treatment algorithms, and ultimately improve the quality of care for ADHF patients in acute care settings. Future research endeavors should focus on validating these findings in diverse patient populations and exploring novel therapeutic strategies aimed at mitigating disease progression and improving survival in ADHF.

## Figures and Tables

**Table 1:** Comparison of parameters (demographic, hematological and biochemical, and categorical) between survivor and exitus groups.

Variable		Survivor N = 163 (71.8%)		Exitus N = 64 (28.2%)		*p*-Value
*Demographic*		Mean ± SD	Median	Mean ± SD	Median	
AGE		63.66 ± 8.10	63.00	66.75 ± 8.88	68.00	0.004 **
*Hematological and Biochemical*		Mean ± SD	Median	Mean ± SD	Median	
HGB (g/dL)		13.88 ± 1.71	14.00	12.25 ± 2.39	12.35	<0.001 **
LY (10^9^/L)		2.98 ± 1.62	2.52	1.98 ± 1.54	1.40	<0.001 **
PLT (10^9^/L)		269.69 ± 79.44	265.00	240.02 ± 92.73	224.50	0.004 **
ALBUMIN (mg/dL)		40.89 ± 3.30	41.00	34.07 ± 6.81	34.00	<0.001 **
HALP SCORE		6.60 ± 3.74	6.03	3.78 ± 3.49	2.68	<0.001 **
*Categorical*		N (%)		N (%)		
GENDER	Female	50 (30.7)		26 (40.6)		0.153
	Male	113 (69.3)		38 (59.4)		
DM	Yes	89 (54.6)		34 (53.1)		0.841
	No	74 (45.4)		30 (46.9)		
HT	Yes	107 (65.6)		51 (79.7)		0.038 *
	No	56 (34.4)		13 (20.3)		
CAD	Yes	85 (52.1)		39 (60.9)		0.231
	No	78 (47.9)		25 (39.1)		
EF (%)	>50	55 (33.7)		22 (34.4)		0.934
	40–50	42 (25.8)		15 (23.4)		
	<40	66 (40.5)		27 (42.2)		

SD: standard deviation; *: difference is significant at the 0.05 level (two-tailed); **: difference is significant at the 0.01 level (two-tailed). HGB: hemoglobin; LY: lymphocyte; PLT: platelet; DM: diabetes mellitus; HT: hypertension; CAD: coronary artery disease; EF: ejection fraction.

**Table 2:** Comparison of parameters (demographic, hematological and biochemical, and categorical) between survivor and exitus groups.

	AUC	*p*-Value	Cut-Off	Sensitivity	Specificity
HEMOGLOBIN	0.714	<0.001	12.95	0.718	0.641
LYMPHOCYTE	0.709	<0.001	1.33	0.902	0.500
PLATELET	0.624	0.003	256.50	0.552	0.672
ALBUMIN	0.814	<0.001	36.70	0.933	0.687
HALP SCORE	0.765	<0.001	3.305	0.840	0.641

AUC: area under curve.

## Data Availability

Data are available in Supplementary Material Table S1.

## Supplementary Materials

The following supporting information can be downloaded at: https://www.mdpi.com/article/10.3390/medicina60091385/s1, Table S1: Dataset.
